# Nearly Complete Genome Sequence of Enterovirus Type A119 from Sewage in France in 2015

**DOI:** 10.1128/mra.00325-23

**Published:** 2023-05-22

**Authors:** Maxime Bisseux, Audrey Mirand, Jonathan Colombet, Jean-Luc Bailly, Cécile Henquell

**Affiliations:** a CHU Clermont-Ferrand, 3 IHP, Centre National de Référence des Entérovirus et Parechovirus, Laboratoire de Virologie, Clermont-Ferrand, France; b Université Clermont Auvergne, LMGE CNRS 6023, UFR de Médecine et des Professions Paramédicales, Clermont-Ferrand, France; Katholieke Universiteit Leuven

## Abstract

A nearly complete genome sequence of enterovirus type A119 was determined from an urban wastewater sample collected during a surveillance campaign in 2015 in Clermont-Ferrand, France. The partial VP1 sequence is a close relative of other partial enterovirus type A119 sequences detected in France and South Africa in the same year.

## ANNOUNCEMENT

Enterovirus (EV) type A119 (EV-A119) belongs to *species A* of the genus *Enterovirus* (family *Picornaviridae*). The virus was first detected in 2008 in the feces of nonhuman primates in Cameroon (1, 2). Later, it was detected in stools of healthy or symptomatic patients with acute flaccid paralysis or diarrhea and in sewage samples (3, 4).

Between 2014 and 2015, we conducted wastewater-based surveillance of EV infections in a sewage treatment plant (Clermont-Ferrand, France) using amplicon-based deep sequencing (5, 6). We detected EV-A119 and reported partial VP1 gene sequences. In the present study, we report a nearly complete EV-A119 genome sequence (designated EV-A119_Wastewater_06/01/2015_CFD_France) obtained from concentrated wastewater samples stored at −20°C.

Total nucleic acids were extracted from 200 μL of stored wastewater using the NucliSENS easyMAG platform (bioMérieux, Marcy l’Etoile, France). Reverse transcription was performed with SuperScript III reverse transcriptase (Invitrogen, Cergy-Pontoise, France) and the reverse primers listed in Table 1, as indicated by the manufacturer. Four genome regions were targeted for gene amplifications and subjected to seminested reactions (PCR1 and PCR2) with the primers listed in Table 1, the Platinum SuperFi I enzyme (Invitrogen) according to the manufacturer’s recommendations, and the reaction conditions indicated in Table 1. The amplicons were purified in a reaction mixture containing DNA and ExoSAP (5:1 [vol/vol]; Life Technologies, Villebon-sur-Yvette, France) for 15 min at 37°C and 15 min at 80°C. Fourteen sequencing reactions were performed with the sequencing primers (Table 1), using the BigDye terminator cycle sequencing kit (Applied Biosystems, Courtaboeuf, France). The sequencing mixtures (final volume of 10 μl) included 3 μl of the purified amplicon, 4 μl of the BigDye Terminator reaction mixture, and 1 μl of the primer solution (10 μM). The sequencing reactions were performed as follows: denaturation at 96°C for 3 min and 25 cycles consisting of denaturation at 96°C for 10 s, annealing at 50°C for 5 s, and elongation at 60°C for 4 min. After purification with NucleoSEQ columns (Macherey-Nagel, Hoerdt, France), sequencing products were analyzed on the 3500 Dx Genetic Analyzer system (Applied Biosystems). The consensus sequence was obtained by assembling the 14 overlapping amplicons using BioEdit v7.1.9. The mean length of the overlaps between two consecutive amplicons was 269 nucleotides. The final genome had a length of 7,432 nucleotides and a GC content of 44.4%. All tools were run with default parameters unless otherwise specified.

**TABLE 1:** Primers used for reverse transcription, amplification, and sequencing of the EV-A119 complete genome

Primer designation	Primer sequence (5′ to 3′)^*a*^	Orientation	Gene target	Usage^*b*^	PCR conditions
IFT7R5NC	GACAGCTTATCATCGTAATACGACTCACTATAGGGTTAAAACAGCCTGTGGGTTG	Forward	P1	PCR1, PCR2, and sequencing	PCR1: hybridization at 60°C, elongation for 2 min 25 s; PCR2: hybridization at 62°C, elongation for 2 min 20 s
5NCS663	GCGGAACCGACTACTTTGGGTGTCCGTGTTTC	Forward	P1	Sequencing	
5NC642B	TGGATTGGCCAYCCRGTG	Reverse	P1	Sequencing	
HEVS436	GGNTGGTRNTGGAANYTNCCNGATG	Forward	P1	Sequencing	
EVA119_1D_R2	GAAGTGAGAAACTGARGAYTCTGCAAGRC	Reverse	P1	PCR2 and sequencing	
EVA119_1D_R1	CTGYTTGTGGAGCATTACCATTCAATCC	Reverse	P1	Reverse transcription and PCR1	
EVA119_1D_S1	GYCTTGCAGARTCYTCAGTTTCTCACTTC	Forward	P1-P2	PCR1	PCR1: hybridization at 65°C, elongation for 1 min 30 s; PCR2: hybridization at 65°C, elongation at 1 min 20 s
EVA119_1D_S2	GGATTGAATGGTAATGCTCCACAARCAG	Forward	P1-P2	PCR2 and sequencing	
HEVAR2C	CGGTGYTTGCTCTTGAACTGCATG	Reverse	P1-P2	Reverse transcription, PCR1, PCR2, and sequencing	
EVA119_2A_S1	CAGGGACCAGGCATCACTGAAGTCC	Forward	P2-P3	PCR1	PCR1: hybridization at 58°C, elongation for 2 min; PCR2: hybridization at 58°C, elongation for 2 min
EVA119_2A_S2	GATACCAGACCCATGTTCTTCTTGGGC	Forward	P2-P3	PCR2 and sequencing	
HEVAS2C	CATGCAGTTCAAGAGCAARCACCG	Forward	P2-P3	Sequencing	
HEVAS_3CD_5572	CCNARCATDGTRAARTGNCCYTG	Forward	P2-P3	Sequencing	
HEVAR_3CD_5572	CCNARCATDGTRAARTGNCCYTG	Reverse	P2-P3	Sequencing	
HEVAR_3CD_6342	CCRTARCANGCNTCYTCCATRCTC	Reverse	P2-P3	Reverse transcription, PCR1, and PCR2	
EVA119_3D_S1	CCAGAACAAATTGAGGGCTCTCC	Forward	P3	PCR1 and sequencing	PCR1: hybridization at 66°C, elongation for 50 s; PCR2: hybridization at 64°C, elongation for 35 s
EVA119_3D_S2	GATTATATGATCCAGGCTGCAAACC	Forward	P3	PCR2	
EVC_3D_S1	GATCCHAGRAAYACNCARGAYC	Forward	P3	Sequencing	
EVA_3NC_02_variant	CYACTGGGTTAGGGTAATTRAYCTTTGG	Reverse	P3	Reverse transcription, PCR1, PCR2, and sequencing	

a Primers were designed for this study based on alignment of all complete genomes of *species A* EVs available in GenBank for generic primers and on our partial sequence of EV-A119 for specific primers (with EVA119 in their designations).

b PCR1 and PCR2, gene amplification reaction rounds 1 and 2, respectively.

**FIG 1:**
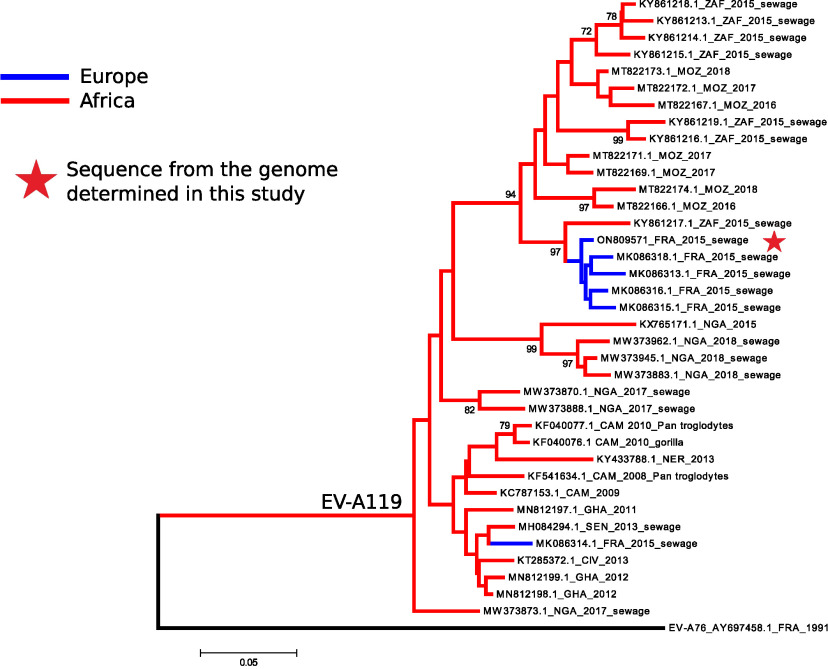
Genetic analysis of an EV-A119 genome sequence. The 37 partial EV-A119 VP1 gene sequences available in GenBank were aligned using BioEdit v7.1.9 and ClustalW (alignment length, 340 nucleotides). The phylogenetic tree was constructed with MEGA5 software (7), using the neighbor-joining method, the Tamura-Nei distance model, and 1,000 bootstrap replicates. Bootstrap values of <70% were hidden. The tree was rooted with the VP1 sequence of an EV-A76 isolate. The branches are colored according to the geographic origin of the sequences (European countries in blue and African countries in red). The VP1 sequence from the nearly complete genome reported in this study is marked with a red star. The countries are indicated with 3-letter ISO 3166-1 alpha-3 codes. Sequences determined from nonhuman specimens are annotated accordingly.

A phylogenetic tree reconstructed with the 37 partial EV-A119 VP1 gene sequences available in GenBank (as of 26 January 2023) showed that 5 of 6 sequences recovered in France, including that determined in this study, clustered consistently with a sequence sampled from wastewater in South Africa the same year (Fig. 1). Another sequence from France was distantly related (Fig. 1). The detection of EV-A119 in wastewater in France in 2015 shows its circulation out of Africa and its ability to cause epidemics locally, as was also described in Nigeria in 2017 and 2018 (3).

### Data availability.

The whole-genome sequence of EV-A119 has been deposited in GenBank under accession number ON809571. The version described in this paper is the first version. The raw data are available in the Sequence Read Archive (SRA) under SRA accession number SRR23606188, BioProject accession number PRJNA938274, and BioSample accession number SAMN33429612.
